# Asian Americans & chronic kidney disease in a nationally representative cohort

**DOI:** 10.1186/s12882-018-1145-5

**Published:** 2019-01-09

**Authors:** Merle Kataoka-Yahiro, James Davis, Krupa Gandhi, Connie M. Rhee, Victoria Page

**Affiliations:** 1 Department of Nursing, School of Nursing and Dental Hygiene, University of Hawai’i at Manoa, 2528 McCarthy Mall, Webster Hall 409, Honolulu, HI 96822 USA; 2 Office of Biostatistics and Quantitative Health Sciences, John A. Burns School of Medicine, Honolulu, HI 96813 USA; 3 Division of Nephrology and Hypertension, University of California Irvine School of Medicine, 101 The City Drive South, City Tower, Suite 400, Orange, CA 92868 USA; 4 National Kidney Foundation of Hawaii, Health Innovation Division, 1314 S. King Street #1555, Honolulu, HI 96814 USA

## Abstract

**Background:**

There is a paucity of specific data on early stages of chronic kidney disease (CKD) among Asian Americans (AAs). The objective of this study was to examine the independent association of Asian race/ethnicity and socio-demographic and co-morbidity factors with markers of early kidney damage, ascertained by ACR levels, as well as kidney dysfunction, ascertained by eGFR levels in a large cross-sectional sample of AAs enrolled in the National Health and Nutrition Examination Survey (NHANES).

**Methods:**

Secondary data analyses of the NHANES 2011–2014 data of a nationally representative sample of 5907 participants 18 years and older, US citizens, and of Asian and White race. NHANES data included race (Asian vs. White), as well as other socio-demographic information and comorbidities. Urine albumin-to-creatinine ratio (ACR) categories and estimated glomerular filtration rate (eGFR) were used as indicators for CKD. Descriptive analyses using frequencies, means (standard deviations), and chi-square tests was first conducted, then multivariable logistic regression serial adjustment models were used to examine the associations between race/ethnicity, other socio-demographic factors (age, sex, education), and co-morbidities (obesity, diabetes, hypertension) with elevated ACR levels (A2 & A3 – CKD Stages 3 and 4–5, respectively) as well as reduced eGFR (G3a-G5 and G3b –G5 - CKD Stage 3–5).

**Results:**

AAs were more likely than White participants to have ACR levels > 300 mg/g (A3) (adjusted OR (aOR) (95% CI) 2.77 (1.55, 4.97), *p* = 0.001). In contrast, adjusted analyses demonstrated that AAs were less likely to have eGFR levels < 60 ml/min/1.73 m2 (G3a-G5) (aOR (95% CI) 0.50 (0.35, 0.72), *p* < .001).

**Conclusions:**

This is one of the first large U.S. population-based studies of AAs that has shown a comparatively higher risk of elevated ACR > 300 mg/g levels (A3) but lower risk of having eGFR levels < 60 ml/min/1.732 m2 (G3a-G5). The findings support the need to address the gaps in knowledge regarding disparities in risk of early stage CKD among AAs.

**Electronic supplementary material:**

The online version of this article (10.1186/s12882-018-1145-5) contains supplementary material, which is available to authorized users.

## Background

Asian Americans (AA)s are projected to be the second fastest growing racial/ethnic group in the U.S and are projected to nearly double to 9.3% of the total population by 2060 [1]. Currently, AAs represent 5.8% of the overall U.S. population [2] and there are approximately 20.4 million Asian adults and children living in the U.S. [3, 4]. Furthermore based on the 2016 U.S. Census, major Asian subgroups of people reported were Chinese (except Taiwanese) (4.9 million), Asian Indian (4.1 million), Filipino (3.9 million), Vietnamese (2.1 million), Korean (1.8 million), and Japanese (1.5 million).

Thirty million adults in the United States (US) have chronic kidney disease (CKD) [5]. Compared to Whites, the prevalence of end-stage renal disease is 1.5 times greater for AAs [6]. AAs constitute about 5.5% of all patients in the U.S. receiving dialysis [7] and 5% of patients living with a functioning kidney transplant in 2013 [8]. In 2011, total Medicare spending rose 5% to $549.1 billion, while end-stage-renal disease expenditures rose 5.4% to $34.3 billion. In addition, total fee-for-service Medicare expenditures per person per year were $87,945 in 2011 [9]. The costs and spending will continue to increase based on the projected population growth of AAs in the U.S. in the next 50 years. Given these projections, disease prevention are imperative to address in the early stages of CKD among this population. While state-level data exists on end-stage renal disease and its treatment, there are no granular and precise data on early stages of CKD specifically among AAs.

CKD is common in people with cardiovascular disease (CVD), diabetes mellitus (DM), hypertension (HTN), and obesity [10]. DM and HTN are the two major risk factors for CKD worldwide [11] and are listed as the primary causes for 70% of new cases of CKD in the U.S. [12]. The prevalence of DM is approximately 40% higher in AAs relative to Whites [12] and 19% of AAs have HTN [13, 14]. Obesity is disproportionately more prevalent in certain Asian subgroups, is also associated with increased risk of development of CKD [15–17] as well as various kidney disease risk factors (e.g., HTN, DM, and dyslipidemia) [18].

There is a paucity of studies exploring the association of albumin to creatinine ratio (ACR) and estimated glomuerular filtration rate (eGFR) with CKD among AAs. International researchers who studied ACR and eGFR with CKD among Asians were from countries such as China, Korea, Japan, and Thailand. Among Asian populations with DM, CKD progresses twice as rapidly [19]. HTN has been associated with CKD among Chinese, Japanese, Filipino, and South Asians [14, 20, 21]. International researchers also found that obesity led to CKD through both indirect (i.e., DM, HTN, dyslipidemia) and direct mechanisms (i.e., glomerular hyperfiltration, inflammation) [22, 23].

Jolly et al. [24] and Mau et al. [25] utilized the U.S. National Kidney Foundation (NKF) Kidney Early Evaluation Program (KEEP) cross-sectional data of community-dwelling racial/ethnic participants and found AAs to have one of the highest odds for CKD based ACR levels. Kataoka-Yahiro et al. [26] and Wong et al. [27] conducted a cross-sectional study of the National Kidney Foundation of Hawaii (NKFH) Kidney Early Screening Program (KEDS) of community dwelling participants to further examine the relationship of risk factors and CKD of Asian Pacific Islanders (Native Hawaiians, Japanese, Chinese, Filipino, and Whites) in Hawaii. ACR and/or urine albumin levels, respectively were used as predictors for CKD. Significant results related to ACR in these studies included BMI, glucose, HTN, and Asian and/or Pacific Islander race/ethnicity.

Racial/ethnic differences in ACR and eGFR evaluation and CKD definition and staging among AAs is particularly needed in the early stages of CKD [28, 29]. While impaired ACR and eGFR have been associated with heightened risk of cardiovascular complications and mortality among Asians [30], further research is needed to determine the impact of ACR and eGFR markers of kidney damage and kidney function, respectively, in AA populations.

Hence, identification of CKD in its earlier stages is a high priority in the AA population [27]. Thus, to better inform the field, we sought to examine the relationship between Asian race/ethnicity and CKD indicators among a nationally representative cohort of AA adults. We examined the independent association of Asian race/ethnicity and socio-demographic and co-morbidity factors with markers of early kidney damage, ascertained by higher ACR levels, as well as kidney dysfunction, ascertained by lower eGFR levels. We further examined the association between socio-demographic and co-morbidity factors with these outcomes stratified by race/ethnicity.

## Methods

### Study design and population

In this study, we examined data from participants enrolled in the National Health and Nutrition Examination Survey (NHANES) [31] over the period of 2011–2014. NHANES is a national population-based survey, designed to assess the health and nutritional status of adults and children in the U.S. The survey is unique in that it combines interviews, physical examinations and laboratory data. In the NHANES study, survey data is obtained via personal household interviews of a stratified, multistage probability sample of the civilian, non-institutionalized population of the U.S. Data from the NHANES were de-identified and provided as downloadable public-use data files.

Participants included were 18 years and older and of AA or non-Hispanic White race/ethnicity. Participants were excluded if they were pregnant women, if their race was Mexican-American or other Hispanic categories, non-Hispanic Black, if they were multi-racial, if they did not identify their race/ethnicity, and if their sampling weights were missing or zero. After the exclusions, the sample included 5907 non-Hispanic Asian and non-Hispanic White participants (referred to in the remainder of the study as Asian and White participants, respectively).

### Measurements and definitions

The primary predictor of interest was race/ethnicity, which was categorized in analyses as Asian and White. In the NHANES study, race/ethnicity information was self-reported by participants. Other predictors included were age, sex, education (measured for SES), DM, HTN, and BMI.

The primary outcomes of interest were ACR and eGFR [9]. ACR was analyzed at two cutpoints: (1) ≥ 30 mg/g (Kidney Disease Improving Global Outcomes [KDIGO] category A2 – CKD Stage 3), and (2) > 300 mg/g (KDIGO category A3 – CKD Stages 4 & 5) [32]. ACR was calculated as urine albumin (mg/dL) divided by urine creatinine (g/dL ratio). In the NHANES study, ACR specimens (urine albumin and urine creatinine) were measured in a random urine collection by the Mobile Examination Center (MEC) (first collection) and a first morning void urine collected by the participant at home (second collection). eGFR was examined at two thresholds chosen as (1) < 60 ml/min/1.73m^2^ (KDIGO category G3a – G5 - Stages 3–5), and (2) < 45 ml/min/1.73m^2^ (KDIGO category G3b –G5 - Stage 3–5). We specifically selected these cut-points as indicators of moderate vs. severe CKD, respectively. The eGFR was calculated using the Chronic Kidney Disease Epidemiology Collaboration equation: eGFR = 141 x min (S_cr_/k,1)^α^ x max (S_cr_/k,1)^-1.209^ × 0.993^Age^ × 1.018 [if female], where, S_cr_ is serum creatinine in mg/dl, k is 0.7 for females and 0.9 for males, α is − 0.329 for females and − 0.411 for males, min indicates the minimum of S_cr_/k or 1, and max indicates maximum of S_cr_/k or 1 [33].

### Statistical analyses

Descriptive statistics for the survey data were summarized by frequencies and percentages for categorical variables, and by means and standard deviations or medians and interquartile ranges for continuous variables, depending on the distributions. Bivariate analyses used Rao-Scott chi-square tests to assess associations between the outcomes ACR and eGFR and other factors.

Multivariable logistic regression analyses were used to examine the association between race/ethnicity, other socio-demographic factors, and comorbidities with elevated ACR, and reduced eGFR, based upon the chosen cut points. The analyses accounted for confounding by age (categorized as 18–64, 65–74, 75 and older years), education (≤ high school/GED, some college, college graduate), sex (male, female), BMI (categorized as normal [< 25.0 kg/m^2^], overweight [25.0–29.9 kg/m^2^] and obesity [≥30.0 kg/m^2^]), and presence of self-reported DM (yes or no) and HTN (yes or no). In secondary analyses, logistic regression models were fit stratified by race/ethnicity using the cut-points for ACR ≥30 mg/g and > 300 mg/g and for eGFR < 60 and <  45 ml/min/1.73m^2^. Results are presented as odds ratios (ORs) with 95% confidence intervals (CIs).

All analyses accounted for the NHANES’s complex multistage sampling design in order to ensure that calculated estimates were representative of the U.S. civilian non-institutionalized population. A value of *p* < 0.05 was considered statistically significant. Statistical analyses were conducted using SAS software, version 9.4 [34]. Figures were generated using R version 3.0.2.

## Results

### Study population

After applying the eligibility criteria, the final study population comprised 5907 Asian and White participants. The total sample size for the ACR analyses was 5792 (115 participants were missing ACR data), and 5605 for the eGFR analyses (302 participants were missing eGFR data). Table 1 provides participants’ descriptive characteristics stratified by race/ethnicity. Asians were more likely than Whites to be 18–64 years of age (86% vs. 79%), college graduates (49.9% vs. 34.4%), have normal weight (60% vs. 31%) and less frequently had HTN (22% vs 34%). Descriptive statistics by race/ethnicity for the outcomes, ACR and eGFR are summarized in Table 2. Asians had higher mean and median levels of ACR than Whites when compared for ACR overall and when examined at A2 and A3 (CKD Stages 3 and 4–5, respectively), ACR levels were skewed towards high values rendering means much higher than medians; thus the median comparisons may be preferable. eGFR levels were reasonably normally distributed and means and medians were similar. Asians has a higher mean overall but the Asian and White means were comparable at levels of G3a–G5 and G3b-G5 (CKD Stage 3–5).

**Table 1:** Characteristics of the study participants by race/ethnicity

Characteristics	Overall (*n* = 5907)	Race (%)		Weighted *p*-value
		Asian (*n* = 1443)	White (*n* = 4464)	
Age Group (years)				< 0.001
18–64	79	86	79	
65–74	12	10	12	
75 and older	9	4	9	
Education Group				< 0.001
≤ High School/GED	30.8	26.6	31.1	
Some College	33.7	23.4	34.5	
College Graduate	35.5	49.9	34.4	
Sex				0.13
Male	49	47	49	
Female	51	53	51	
Body Mass Index (kg/m^2^)				< 0.001
Normal	34	60	31	
Overweight	33	28	34	
Obese	33	12	35	
Have Hypertension				< 0.001
Yes	34	22	34	
No	66	78	66	
Have Diabetes				0.92
Yes	11	11	11	
No	89	89	89	

Weighted column percentage. Analyses conducted using chi-square tests

**Table 2:** Mean and median urine albumin-to-creatinine ratio (ACR) and estimated glomerular filtration rate (eGFR) by race

		Race	
Outcome	Statistic	Asians	White
ACR mg/g (A1, A2, A3)	Mean ± SE	38.4 ± 7.1	25.7 ± 2.6
[CKD Stage 1–5]	Median (Q1, Q3)	7.2 (4.8, 12.8)	6.8 (4.6, 12.1)
ACR ≥ 30 mg/g (A2)	Mean ± SE	311.4 ± 68.1	201.4 ± 27.0
[CKD Stage 3]	Median (Q1, Q3)	76.6 (39.8, 198.1)	57.4 (39.6, 120.8)
ACR > 300 mg/g (A3)	Mean ± SE	1255.7 ± 298	1225.1 ± 204
[CKD Stages 4–5]	Median (Q1, Q3)	628.2 (427.0, 1081.6)	657.1 (404.9, 1140.9)
eGFR ml/min/1.73 m2 (G1 to G5)	Mean ± SE	101.2 ± 0.5	90.3 ± 0.3
[CKD Stages 1–5]	Median (Q1,Q3)	103.3 (90.2, 115.5)	91.8 (76.8, 105.5)
eGFR < 60 ml/min/1.73 m2 (G3a-G5)	Mean ± SE	48.6 ± 1.63	47.7 ± 0.57
[CKD Stage 3 to 5]	Median (Q1, Q3)	51.8 (43.9, 57.5)	50.7 (41.9, 55.7)
eGFR < 45 ml/min/1.73 m2 (G3b-G5)	Mean ± SE	34.1 ± 2.8	33.5 ± 0.9
[CKD Stage 3 to 5]	Median (Q1, Q3)	37.1 (27.9, 42.6)	35.7 (30.6, 41.0)

*SE* standard error; *Q1* 1st quartile, *Q3* 3rd quartile

Note: UACR has a skewed distribution and therefore we would suggest using median (Q1, Q3), while eGFR is normally distributed and therefore would suggest using mean and standard error

ACR ≥ 30 mg/g (A2) – Moderately increased; ACR > 300 mg/g (A3) – Severely increased; eGFR < 60 ml/min/1.73 m2 (G3a) – Mildly to moderately decreased; eGFR < 45 ml/min/1.73 m2 (G3b) – Moderately to severely decreased

### Comparison of Asians and whites in serially-adjusted, multivariable logistic regression models for ACR ≥ 30 mg/g and ACR > 300 mg/g (A2 and A3) and eGFR < 60 ml/min/1.73 m^2^ and eGFR < 45 ml/min/1.73 m^2^ (G3a-G5 and G3b-G5)

**Fig. 1:**
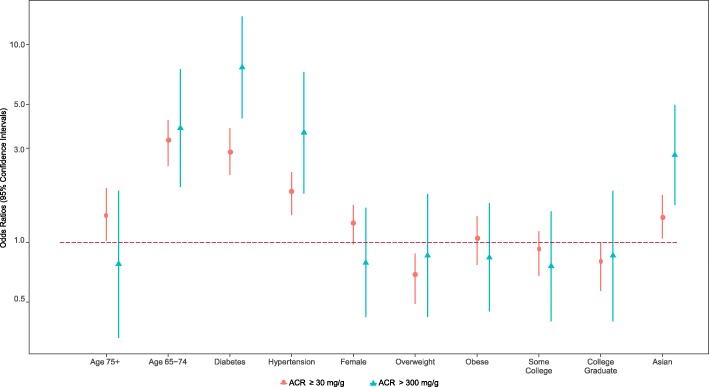
Comparison of Asians & Whites in Multivariable Logistic Regression Models Adjusted for ACR ≥30 mg/g (A2) and ACR > 300 mg/g (A3)

**Fig. 2:**
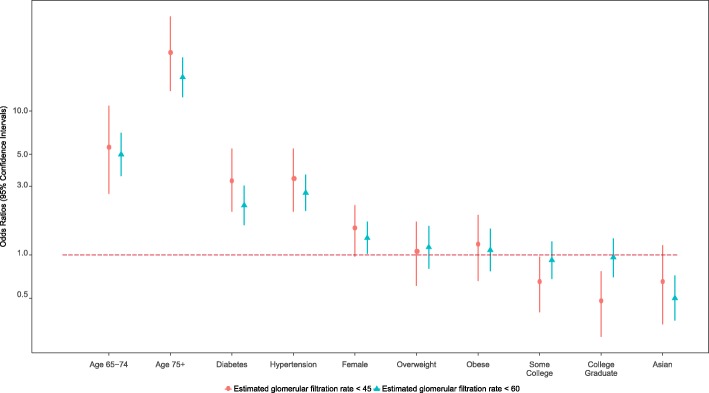
Comparison of Asians & Whites in Adjusted Multivariable Logistic Regression Models for eGFR < 60 mg/ml/1.73 m2 and eGFR < 45 mg/ml/1.73 m2

The ACR levels of Asians and Whites were compared using four models of serial adjustments: unadjusted; age- and sex-adjusted; age-, sex- and education adjusted, including education as a measure of socioeconomic status; and a model with additional adjustment for health characteristics (normal, overweight, obesity, DM, and HTN). For ACR levels ≥30 mg/g differences were not significant in the unadjusted model, but with age-, sex-adjustment and with further adjustment, Asians had increasing odds of higher ACR levels than Whites at 1.35 (95% CI = 1.05, 1.74, weighted *p*-value = 0.020) (Table 3; Fig. 1). Significant socio-demographic and co-morbidity factors were (a) age 65–74 and 75 and older with odds ratios of 1.39 and 3.19, (95% CI = 1.02, 1.89; 2.43, 4.17), respectively; (b) BMI overweight with an odds ratio of − 0.65 (95% CI = 0.49, 0.88); (c) DM with an odds ratio of 2.89 (95% CI = 2.20, 3.79); and (d) HTN with an odds ratio of 1.77 (95% CI = 1.38, 2.27). Using ACR of > 300 mg/g, Asians had significantly higher odds in all of the serial-adjusted models reaching an odds ratio of 2.77 in the fully adjusted model (95% CI = 1.55, 4.97, weighted *p*-value = 0.001). Significant socio-demographic and co-morbidity factors were (a) age 75 and older with an odds ratio of 3.79 (95% CI = 1.91, 7.52); (b) DM with odds ratio of 7.68 (95% CI = 4.24, 13.92); and (c) HTN with odds ratio of 3.6 (95% CI = 1.77, 7.29) (Table 3; Fig. 1). At the eGFR level of < 60 ml/min/1.73 m^2^, Asians consistently had lower odds compared to Whites in all of the serial adjusted models. The odds ratio went from 0.38 (95% CI = 0.27–0.52, weighted *p*-value = < 0.001) in the unadjusted model to 0.50 (95% CI = 0.35, 0.72, weighted *p*-value = < 0.001) in the fully adjusted model (Table 4; Fig. 2). Significant socio-demographic and co-morbidity factors were (a) ages 65–75 and 75 and older with odds ratios of 4.99 and 17.15 (95% CI = 3.53, 7.06 and 12.45, 23.61), respectively; (b) DM with an odds ratio of 2.21 (95% CI = 1.61, 3.03); and (c) HTN with an odds ratio of 2.7 (95% CI = 2.02, 3.61). For eGFR < 45 ml/min/1.73m^2^, Asians had a significantly lower odds than Whites in the unadjusted model [odds ratio = 0.39 (95% CI = 0.22, 0.67, weighted *p*-value = 0.001)] but the odds ratio increased and became non-significant with age-, sex- adjustment, and remained non-significant with adjustment for additional covariates [odd ratio = 0.62 (95% CI = 0.33, 1.17, weighted *p*-value = 0.139) (Table 4: Fig. 2).

**Table 3:** Comparison of Asians and Whites in serially-adjusted, multivariable logistic regression models for ACR ≥ 30 mg/g (A2) and ACR > 300 mg/g (A3)

		ACR ≥ 30 mg/g (A2)		ACR > 300 mg/g (A3)	
Regression Model	Comparisons	Odds Ratio (95% CI)	Weighted *p*-value	Odds Ratio (95% CI)	Weighted *p*-value
Model 1	Asian vs White	1.12 (0.9, 1.39)	0.301	2.06 (1.25, 3.39)	0.005
Model 2	Ages 65–74 vs Ages < 65	2.01 (1.5, 2.68)	0.000	1.86 (0.81, 4.28)	0.144
	Ages 75 & higher vs Ages < 65	4.49 (3.52, 5.72)	0.000	8.54 (4.58, 15.92)	0.000
	Female vs Male	1.25 (1, 1.55)	0.050	0.75 (0.42, 1.35)	0.342
	Asian vs White	1.27 (1.01, 1.58)	0.040	2.62 (1.53, 4.47)	0.000
Model 3	Ages 65–74 vs Ages < 65	1.98 (1.48, 2.65)	0.000	1.83 (0.79, 4.21)	0.157
	Ages 65–74 vs Ages < 65	4.26 (3.33, 5.44)	0.000	8.03 (4.39, 14.68)	0.000
	Female vs Male	1.24 (0.99, 1.54)	0.059	0.74 (0.41, 1.34)	0.322
	Some College vs < HS/GED	0.86 (0.67, 1.11)	0.252	0.77 (0.41, 1.45)	0.416
	College Graduate vs < HS/GED	0.65 (0.49, 0.85)	0.002	0.64 (0.31, 1.29)	0.211
	Asian vs White	1.32 (1.05, 1.66)	0.017	2.7 (1.55, 4.7)	0.000
Model 4	Ages 65–74 vs Ages < 65	1.39 (1.02, 1.89)	0.038	0.78 (0.33, 1.83)	0.563
	Ages 75 & higher vs Ages < 65	3.19 (2.43, 4.17)	0.000	3.79 (1.91, 7.52)	0.000
	Female vs Male	1.23 (0.98, 1.55)	0.072	0.79 (0.42, 1.5)	0.473
	Some College vs < HS/GED	0.88 (0.68, 1.14)	0.328	0.76 (0.4, 1.44)	0.400
	College Graduate vs < HS/GED	0.76 (0.57, 1)	0.051	0.86 (0.4, 1.83)	0.694
	BMI (Overweight vs Normal)	0.65 (0.49, 0.88)	0.005	0.86 (0.42, 1.76)	0.673
	BMI (Obese vs Normal)	1.02 (0.77, 1.36)	0.870	0.84 (0.45, 1.58)	0.595
	Diabetes (Yes vs No)	2.89 (2.2, 3.79)	0.000	7.68 (4.24, 13.92)	0.000
	Hypertension (Yes vs No)	1.77 (1.38, 2.27)	0.000	3.6 (1.77, 7.29)	0.000
	Asian vs White	1.35 (1.05, 1.74)	0.020	2.77 (1.55, 4.97)	0.001

*CI* confidence interval, *BMI* body mass index, *< HS/GED* less than high school or GED equivalent

ACR ≥ 30 mg/g (A2) – Moderately increased; ACR > 300 mg/g (A3) – Severely increased

**Table 4:** Comparison of Asians and Whites in serially-adjusted, multivariable logistic regression models for eGFR < 60 ml/min/1.73m^2^ (G3a-G5) and eGFR < 45 ml/min/1.73m^2^ (G3b-G5)

		eGFR < 60 ml/min/1.73m^2^		eGFR < 45 ml/min/1.73m^2^	
Regression Model	Comparisons	Odds Ratio (95% CI)	Weighted *p*-value	Odds Ratio (95% CI)	Weighted *p*-value
Model 1	Asian vs White	0.38 (0.27, 0.52)	0.000	0.39 (0.22, 0.67)	0.001
	Ages 65–74 vs Ages < 65	7.40 (5.3, 10.32)	0.000	9.64 (5.03, 18.5)	0.000
Model 2	Ages 75 & higher vs Ages < 65	25.41 (18.83, 34.27)	0.000	45.26 (25.46, 80.44)	0.000
	Female vs Male	1.25 (0.97, 1.61)	0.082	1.48 (1.02, 2.13)	0.037
	Asian vs White	0.50 (0.35, 0.7)	0.000	0.61 (0.34, 1.09)	0.098
Model 3	Ages 65–74 vs Ages < 65	7.34 (5.26, 10.24)	0.000	9.27 (4.82, 17.85)	0.000
	Ages 75 & higher vs Ages < 65	24.7 (18.33, 33.29)	0.000	40.49 (22.31, 73.47)	0.000
	Female vs Male	1.23 (0.96, 1.59)	0.101	1.36 (0.94, 1.98)	0.101
	Some College vs < HS/GED	0.89 (0.67, 1.2)	0.451	0.64 (0.42, 0.98)	0.039
	College Graduate vs < HS/GED	0.79 (0.58, 1.07)	0.126	0.36 (0.22, 0.6)	0.000
	Asian vs White	0.50 (0.36, 0.71)	0.000	0.63 (0.35, 1.15)	0.134
Model 4	Ages 65–74 vs Ages < 65	4.99 (3.53, 7.06)	0.000	5.38 (2.66, 10.89)	0.000
	Ages 75 & higher vs Ages < 65	17.15 (12.45, 23.61)	0.000	25.06 (13.8, 45.52)	0.000
	Female vs Male	1.31 (1.02, 1.7)	0.038	1.47 (0.97, 2.22)	0.066
	Some College vs < HS/GED	0.92 (0.68, 1.24)	0.576	0.62 (0.4, 0.97)	0.038
	College Graduate vs < HS/GED	0.96 (0.7, 1.3)	0.775	0.46 (0.27, 0.77)	0.004
	BMI (Overweight vs Normal)	1.13 (0.8, 1.59)	0.484	1.02 (0.61, 1.7)	0.944
	BMI (Obese vs Normal)	1.08 (0.77, 1.52)	0.648	1.12 (0.66, 1.9)	0.679
	Diabetes (Yes vs No)	2.21 (1.61, 3.03)	0.000	3.19 (1.99, 5.1)	0.000
	Hypertension (Yes vs No)	2.7 (2.02, 3.61)	0.000	3.31 (2, 5.48)	0.000
	Asian vs White	0.50 (0.35, 0.72)	0.000	0.62 (0.33, 1.17)	0.139

*CI* confidence interval, *BMI* body mass index, <HS/GED less than high school or GED equivalent

eGFR < 60 ml/min/1.73 m2 (G3a-G5) – Mildly to moderately decreased; eGFR < 45 ml/min/1.73 m2 (G3b-G5) – Moderately to severely decreased

### Association of Other Socio-Demographic and Comorbidities with CKD outcomes across racial/ethnic subgroups

To better understand the risks of Asians and Whites, separate models were fit for the two ethnicities. For ACR levels ≥30 mg/g, diabetes (*p* = 0.001) and having hypertension (*p* = .022) were significant risk factors for both Asians and Whites and only sex for Whites (*p* = 0.000) (Additional file 1: Table S1). For ACR levels > 300 mg/g DM was significant risk factor for both ethnicities (*p* = 0.032 and 0.000, respectively). Older age and HTN was also significant for Whites (*p* = 0.000 and *p* = 0.002, respectively) (Additional file 1: Table S2). For eGFR < 60 ml/min/1.73 m^2^, older age (*p* = 0.000), DM (*p* = 0.001 and 0.000, respectively), and having HTN (*p* = 0.003 and 0.000), were significant risk factors for Asians and for Whites (Additional file 1: Table S3). Results had a similar pattern using eGFR < 45 ml/min/1.73 m^2^ although statistical significance was not observed (Additional file 1: Table S4).

## Discussion

The objective of this study was to examine the relationship of Asian race/ethnicity, socio-demographic and co-morbidity factors, and CKD outcomes in a nationally representative sample of AAs and Whites who were enrolled in the NHANES study from 2011 to 2014. To our knowledge, this is one of the first large U.S. population-based studies that has shown a comparatively higher risk of elevated ACR levels (A3), an early marker of kidney damage, among Asians compared to Whites.

CKD is defined as the abnormality of kidney structure or function, present for > 3 months, with varying implications upon health status and CKD Stages 1 to 5 depending upon underlying cause. In this study, the ACR and eGFR levels of Asians and Whites were compared using four models of serial adjustments for socio-demographic and co-morbidity factors, adjusted and unadjusted. With adjustment, ACR was a key determinant of CKD [35, 36]. In this study, socio-demographic and co-morbidity factors which were significant for KDIGO-defined A2, A3, and G3a-G5 were related to age, DM, and HTN independent of race/ethnicity. Similarly, when race/ethnicity was stratified into Asian and White; age, DM, and HTN were significant factors related to A2, A3, and G3a-G5. These results were consistent with the existing literature [24–27]. Other studies also indicated that albuminuria was an important correlate of HTN among patients with CKD [37, 38] as well as an indicator of insulin resistance [39, 40]. Age was the best correlate of low eGFR (eGFR < 60 ml/min/1.73m^2^) while HTN was the greatest predictor of albuminuria [41]. In this study, obesity was a significant independent factor worth mentioning for A2, but not for A3 or eGFR of 60 ml/min/1.73m^2^. There were several studies which support this study finding [42, 43], but obesity was not a consistent finding in this study with ACR or eGFR. In this study, albuminuria and eGFR prevalence was independently higher among participants who were (a) older, (b) with DM, (c) had HTN, and (d) of Asian background. These findings suggest a need for access to early screening and detection of kidney damage in the community [24, 26, 27, 44]. More specifically, targeted programs which include not only CKD early screening, but also encompasses the identification of CVD risk factors (HTN, DM, Obesity) inclusive of culturally appropriate screening, detection, referral, and intervention in routine kidney screening [24, 26].

In this study, we observed that Asian participants had a 2.77-fold higher risk of KDIGO-defined albuminuria categories A3 and a trend towards higher risk of category A2. Studies of AA samples have also similar results of a trend towards higher risk of albuminuria category A2 [24, 26, 27, 45, 46]. There is a paucity of studies which have found a higher risk of albuminuria category A3 among AA participants compared to their non-Hispanic White counterparts. Possible reasons to consider to explain this apparent higher risk may include diet [47], genetic or environmental [45, 48–50], urine specimen procedure [51, 52], and other laboratory values [53, 54]. Sato et al. [47] analyzed 675 men and 924 women who did not have DM and found those who had less animal protein and more B-cryptoxanthin in the diet did not have abnormal microalbuminuria in comparison to those who ingested n-3 polyunsaturated fatty acids. Sakamoto et al. [50] found YKL-40 levels were significantly elevated in type 1 diabetic patients than in healthy controls and demonstrated YKL-40 levels were a determinant of ACR independent of other risk factors. Prior et al. [55] found a significant association between genotype (GG/GA/AA) and urinary albumin (normoalbumuria vs. micro/macroalbuminuria, *p* = 0.01). Mashima et al. [56] single nucleotide polymorphisms (SNPs) in six proinflammatory cytokine genes IL-6 and CCL1 genes were associated with albuminuria and the combination of these genotypes had an additive effect on the prevalence and severity of albuminuria.

Matsushita et al. [51] reported that measurements of creatinine and albuminuria were not standardized. In some studies, researchers measured creatinine and albuminuria using fresh samples while others used frozen samples which may have an effect on the validity of results for both eGFR and albuminuria. Carter et al. [52] described the influence of urine creatinine concentrations on the relation of albumin-creatinine ratio with cardiovascular disease events from the Multi-Ethnic Study of Athersclerosis (MESA). Carter et al. found urine creatinine was lower in older female, and White and Chinese participants, and those with lower body weight (reflecting lower muscle mass). The urine tonicity may make spot urine creatinine a less reliable marker of muscle mass than 24 h urine creatinine.

In this study, we utilized the CKD-EPI Creatinine Equation based on the CKD Prognosis Consortium (CKD-PC) [57–59]. The CKD-PC provided a comprehensive method to define and stage CKD, eGFR, and albuminuria on kidney outcomes.

In our analyses of kidney function defined by eGFR as the outcome of interest, we observed contrasting associations, such that Asians had a lower risk of mild to moderately reduced eGFR (< 60 ml/min/1.73m^2^) compared to Whites. There are limitations in the application of creatinine-based GFR estimating equations. For example, the equation ascertaining kidney function using these methods were not specifically derived from AA populations and cannot overcome the limitations of serum creatinine as a filtration marker, which may be lower in Asian populations due to lower generation of creatinine from muscle. Indeed, several studies have proposed using an Asian-specific ethnic equation for the Chronic Kidney Disease Epidemiology Collaboration formula analysis. Hence, in our study the eGFR findings must be carefully interpreted, and further studies are needed to define what ethnic equation may be used for AAs. A limitation in the use of serum creatinine to eGFR ratio within this study is that the ratio is different among various Asian subgroup populations. For example, Horio et al. [60] and Teo et al. [61] reported the correction factors of Chinese, Malay, and Indians/other (1.100, 1.032, and 0.996 for the CKD-EPI equation, respectively). However, in the NHANES cohort, AAs were examined as a single non-granular sub-group. Levey, Becker, and Inker [33] have recommended for the initial assessment of eGFR, measuring serum creatinine and reporting eGFR be based on CKD-EPI 2009 equation. They also mentioned if there is difficulty to confirm the eGFR results based on “extremes of muscle mass, diet, interference of assay”, cystatin C should be measured and eGFR should be measured directly using clearance procedure rather than initial assessment of albuminuria measuring urine albumin and creatinine based on ‘spot’ urine collection and reporting of ACR (p.1, 5–6). Hence, further studies are needed to determine the role of cystatin C and cystatin-C based eGFR assessment among the AA population and individual subgroups.

Our study has a number of strengths, including its examination of a large nationally represent cohort of healthy U.S. participants with a large sample of AAs; comprehensive availability of detailed information, including socio-demographic and comorbidity data; and rigorous analytic approaches that examined various thresholds of ACR and eGFR and accounted for key confounders of the Asian race/ethnicity - CKD associations. However, several limitations of our study should be acknowledged. First, given the study’s cross-sectional and observational nature, our findings do not confirm causal associations. Second, as socio-demographic and comorbidity data were self-reported by the participants, it is possible that the participants’ responses may be affected by biases. Participants who self-reported and self-selected to be in this study may not be representative of the general population. This study did not report on lab values, medications, or management of HTN, DM, obesity, or CKD which could provide additional objective information along with self-reported data. Third, since AAs were aggregated in the NHANES cohort, the results may not be generalizable to Asian subgroups. Fourth, the definition of eGFR is based on single assessment of serum creatinine which may introduce misclassification bias. Low muscle mass and serum creatinine and creatinine generation in AAs may also affect the denominator in the ACR ratio, thereby falsely augmenting ACR among this population.

## Conclusions

In conclusion, our findings suggest that AAs are at higher risk of early damage manifested by abnormal levels of albuminuria. It is important to continue to explore and examine large cohorts or longitudinal studies on AAs and sub-groups in order to accurately identify CKD prevalence in community dwelling populations. Valid and reliable outcome measures of ACR and eGFR are needed to examine the relationship to socio-demographic and co-morbidity risk factors in efforts to justify a need to target culturally appropriate early screening, detection, referral, and intervention in community settings.

## Additional file

Additional file 1: **Table S1.** Multivariable logistic regression models for urine albumin-to-creatinine ratios (ACR) ≥ 30 mg/g (A2) stratified by race/ethnicity. **Table S2.** Multivariable logistic regression models for urine albumin-to-creatinine ratios (ACR) > 300 mg/g (A3) stratified by race/ethnicity. **Table S3.** Multivariable logistic regression models for eGFR < 60 ml/min/1.73 m2 (G3a-G5) stratified by race/ethnicity. **Table S4.** Multivariable logistic regression models for eGFR < 45 ml/min/1.73 m2 (G3b-G5) stratified by race/ethnicity. (DOCX 18 kb)

## Abbreviations

AA: Asian American
ACR: Urine albumin-to-creatinine ratio
BMI: Body Mass Index
CKD: Chronic Kidney Disease
CVD: Cardiovascular disease
DM: Diabetes mellitus
eGFR: Estimated glomerular filtration rate
HTN: Hypertension
KEDS: Kidney Early Screening Program
KEEP: Kidney Early Evaluation Program
MDRD: Modification of Diet in Renal Disease
NHANES: National Health and Nutrition Examination Survey
OR: Odds ratio
U.S.: United States
