# Supplementary material for: Asian Americans & chronic kidney disease in a nationally representative cohort
Source: BMC Nephrol. 2019 Jan 9;20:10. doi: 10.1186/s12882-018-1145-5 (PMC6327460; doi:10.1186/s12882-018-1145-5)
Supplement: Supplementary file 1 — Table S1. Multivariable logistic regression models for urine albumin-to-creatinine ratios (ACR) ≥ 30 mg/g (A2) stratified by race/ethnicity. Table S2. Multivariable logistic regression models for urine albumin-to-creatinine ratios (ACR) > 300 mg/g (A3) stratified by race/ethnicity. Table S3. Multivariable logistic regression models for eGFR < 60 ml/min/1.73 m2 (G3a-G5) stratified by race/ethnicity. Table S4. Multivariable logistic regression models for eGFR < 45 ml/min/1.73 m2 (G3b-G5) stratified by race/ethnicity. (DOCX 18 kb) [file 12882_2018_1145_MOESM1_ESM.docx]

# Additional file

Additional file 1: Table S1. Multivariable logistic regression models for urine albumin-to-creatinine ratios (ACR) ≥ 30 mg/g (A2) stratified by race/ethnicity

| ACR | Comparisons | Asians  Odds Ratio | Weighted | Whites  Odds Ratio | Weighted |
| --- | --- | --- | --- | --- | --- |
| Level |  | (95% CI) | p-value | (95% CI) | p-value |
| **≥30 mg/g** | Ages 65-74 vs Ages < 65 | 1.44 (0.69, 2.97) | 0.328 | 0.96 (0.59, 1.57) | 0.880 |
| **(A2)** | Ages 75 & higher vs Ages < 65 | 1.56 (0.59, 4.09) | 0.369 | 3.07 (2.11, 4.49) | 0.000 |
|  | Female vs Male | 0.72 (0.43, 1.21) | 0.215 | 1.27 (0.92, 1.77) | 0.150 |
|  | Some College vs < HS/GED | 0.83 (0.42, 1.62) | 0.577 | 0.99 (0.69, 1.43) | 0.957 |
|  | College Graduate vs < HS/GED | 0.56 (0.31, 1.02) | 0.059 | 0.93 (0.61, 1.41) | 0.717 |
|  | BMI (Overweight vs Normal) | 0.86 (0.48, 1.55) | 0.610 | 0.85 (0.54, 1.34) | 0.477 |
|  | BMI (Obese vs Normal) | 1.52 (0.76, 3.06) | 0.239 | 1.02 (0.67, 1.56) | 0.933 |
|  | Diabetes (Yes vs No) | 2.76 (1.53, 4.96) | 0.001 | 3.26 (2.25, 4.71) | 0.000 |
|  | Hypertension (Yes vs No) | 1.98 (1.11, 3.54) | 0.022 | 2.37 (1.61, 3.48) | 0.000 |

CI=confidence interval; BMI=body mass index; < HS/GED = less than high school or GED equivalent

≥ 30 mg/g (A2) – Moderately increased; ACR > 300 mg/g (A3) – Severely increased

Additional file 1: Table S2. Multivariable logistic regression models for urine albumin-to-creatinine ratios (ACR) > 300 mg/g (A3) stratified by race/ethnicity

Asians Whites

ACR Level Comparisons

Odds Ratio (95% CI)

Weighted p-value

Odds Ratio (95% CI)

Weighted p-value

| **≥ 300mg/g** | Ages 65-74 vs Ages < 65 | 4.44 (1.67, 11.85) | 0.003 | 0.5 (0.14, 1.77) | 0.281 |
| --- | --- | --- | --- | --- | --- |
| **(A3)** | Ages 75 & higher vs Ages < 65 | 0.88 (0.1, 7.68) | 0.907 | 3.95 (1.9, 8.22) | 0.000 |
|  | Female vs Male | 0.68 (0.28, 1.64) | 0.388 | 0.78 (0.37, 1.62) | 0.502 |
|  | Some College vs < HS/GED | 0.77 (0.23, 2.62) | 0.675 | 0.74 (0.37, 1.5) | 0.408 |
|  | College Graduate vs < HS/GED | 1.14 (0.47, 2.76) | 0.778 | 0.83 (0.33, 2.09) | 0.693 |
|  | BMI (Overweight vs Normal) | 0.64 (0.23, 1.78) | 0.396 | 0.91 (0.38, 2.17) | 0.828 |
|  | BMI (Obese vs Normal) | 1.61 (0.59, 4.39) | 0.348 | 0.82 (0.4, 1.71) | 0.603 |
|  | Diabetes (Yes vs No) | 2.77 (1.09, 7.01) | 0.031 | 8.93 (4.48, 17.77) | 0.000 |
|  | Hypertension (Yes vs No) | 1.74 (0.67, 4.49) | 0.254 | 4.09 (1.7, 9.83) | 0.002 |

CI=confidence interval; BMI=body mass index; < HS/GED = less than high school or GED equivalent

ACR ≥ 30 mg/g (A2) – Moderately increased; ACR > 300 mg/g (A3) – Severely increased

Additional file 1: Table S3. Multivariable logistic regression models for eGFR < 60ml/min/1.73m2 (G3a-G5) stratified by race/ethnicity eGFR Level

| Comparisons | Asians  Odds Ratio (95% CI) | Weighted p-value | Whites  Odds Ratio (95% CI) | Weighted p-value |
| --- | --- | --- | --- | --- |
| Ages 65-74 vs Ages < 65 | 5.23 (2.35, 11.66) | 0.000 | 4.98 (3.48, 7.11) | 0.000 |
| Ages 75 & higher vs Ages < 65 | 24.5 (10.07, 59.63) | 0.000 | 16.95 (12.22, 23.52) | 0.000 |
| Female vs Male | 0.58 (0.30, 1.15) | 0.121 | 1.35 (1.04, 1.76) | 0.026 |
| Some College vs < HS/GED | 0.77 (0.28, 2.12) | 0.616 | 0.92 (0.68, 1.26) | 0.611 |
| College Graduate vs < HS/GED | 0.72 (0.34, 1.54) | 0.399 | 0.97 (0.70, 1.33) | 0.835 |
| BMI (Overweight vs Normal) | 0.72 (0.32, 1.61) | 0.427 | 1.15 (0.81, 1.65) | 0.437 |
| BMI (Obese vs Normal) | 0.38 (0.09, 1.51) | 0.169 | 1.11 (0.78, 1.58) | 0.551 |
| Diabetes (Yes vs No) | 3.47 (1.68, 7.19) | 0.001 | 2.16 (1.55, 3.00) | 0.000 |
| Hypertension (Yes vs No) | 3.1 (1.45, 6.60) | 0.003 | 2.69 (2.00, 3.62) | 0.000 |

# eGFR <

**60ml/min/1.73m2 (G3a-G5)**

CI=confidence interval; BMI=body mass index; < HS/GED = less than high school or GED equivalent

eGFR < 60 ml/min/1.73m2 (G3a-G5) – Mildly to moderately decreased

| eGFR Level Comparisons | | Asians Odds Ratio (95% CI) | Weighted p-value | Whites Odds Ratio (95% CI) | Weighted p-value |
| --- | --- | --- | --- | --- | --- |
| **eGFR <** | Ages 65-74 vs Ages < 65 | 4.06 (0.86, 19.17) | 0.077 | 5.49 (2.65, 11.39) | 0.000 |
| **45ml/min/1.73m2** | Ages 75 & higher vs Ages < 65 | 20.23 (4.19, 97.68) | 0.000 | 25.31 (13.66, 46.9) | 0.000 |
| **(G3b-G5)** | Female vs Male | 0.58 (0.30, 1.15) | 0.121 | 1.35 (1.04, 1.76) | 0.026 |
|  | Some College vs < HS/GED | 0.77 (0.28, 2.12) | 0.616 | 0.92 (0.68, 1.26) | 0.611 |
|  | College Graduate vs < HS/GED | 0.72 (0.34, 1.54) | 0.399 | 0.97 (0.70, 1.33) | 0.835 |
|  | BMI (Overweight vs Normal) | 0.59 (0.15, 2.31) | 0.453 | 1.04 (0.61, 1.77) | 0.894 |
|  | BMI (Obese vs Normal) | 0.93 (0.18, 4.90) | 0.929 | 1.13 (0.66, 1.96) | 0.652 |
|  | Diabetes (Yes vs No) | 4.57 (1.24, 16.75) | 0.022 | 3.16 (1.94, 5.13) | 0.000 |
|  | Hypertension (Yes vs No) | 4.08 (0.85, 19.68) | 0.080 | 3.28 (1.95, 5.52) | 0.000 |

CI=confidence interval; BMI=body mass index; < HS/GED = less than high school or GED equivalent

eGFR < 45 ml/min/1.73m2 (G3b-G5) – Moderately to severely decreased
